# Behavioral Monitoring Tool for Pig Farmers: Ear Tag Sensors, Machine Intelligence, and Technology Adoption Roadmap

**DOI:** 10.3390/ani11092665

**Published:** 2021-09-10

**Authors:** Santosh Pandey, Upender Kalwa, Taejoon Kong, Baoqing Guo, Phillip C. Gauger, David J. Peters, Kyoung-Jin Yoon

**Affiliations:** 1 Department of Electrical and Computer Engineering, Iowa State University, Ames, IA 50011, USA; upender_kalwa@outlook.com; 2 Center for Defense Acquisition and Requirements Analysis, Korea Institute for Defense Analyses, 37 Hoegi-ro, Dongdaemun-gu, Seoul 02455, Korea; taejoonkong@gmail.com; 3 Department of Veterinary Diagnostic and Production Animal Medicine, Iowa State University, Ames, IA 50011, USA; bqguo@iastate.edu (B.G.); pcgauger@iastate.edu (P.C.G.); 4 Rural Sociology, Department of Sociology and Criminal Justice, Iowa State University, Ames, IA 50011, USA; dpeters@iastate.edu

**Keywords:** precision swine farming, ear tag pig sensor, behavioral monitoring, machine intelligence, technology adoption

## Abstract

**Simple Summary:**

In a pig farm, it is challenging for the farm caretaker to monitor the health and well-being status of all animals in a continuous manner throughout the day. Automated tools are needed to remotely monitor all the pigs on the farm and provide early alerts to the farm caretaker for situations that need immediate attention. With this goal, we developed a sensor board that can be mounted on the ears of individual pigs to generate data on the animal’s activity, vocalization, and temperature. The generated data will be used to develop machine learning models to classify the behavioral traits associated with each animal over a testing period. A number of factors influencing the technology adoption by farm caretakers are also discussed.

**Abstract:**

Precision swine production can benefit from autonomous, noninvasive, and affordable devices that conduct frequent checks on the well-being status of pigs. Here, we present a remote monitoring tool for the objective measurement of some behavioral indicators that may help in assessing the health and welfare status—namely, posture, gait, vocalization, and external temperature. The multiparameter electronic sensor board is characterized by laboratory measurements and by animal tests. Relevant behavioral health indicators are discussed for implementing machine learning algorithms and decision support tools to detect animal lameness, lethargy, pain, injury, and distress. The roadmap for technology adoption is also discussed, along with challenges and the path forward. The presented technology can potentially lead to efficient management of farm animals, targeted focus on sick animals, medical cost savings, and less use of antibiotics.

## 1. Introduction

The economics of a pig farm is dependent on the health and welfare status of pigs [1]. Therefore, all stakeholders in the pig industry want to ensure that pigs display normal behavior and physiological functioning with the absence of lesions, diseases, or malnutrition. This requires frequent assessment of their well-being status and disease symptoms. Good well-being is reached when the animal is in harmony with itself and its environment, whereas poor well-being happens when the animal is exposed to infections and adverse conditions resulting from different management practices. In general, poor well-being is manifested by behavioral changes (e.g., abnormal movement, reduced feeding or drinking, lethargy, or aggressive nature), physiological changes (e.g., increased heart rate or respiration rate), and pathological changes (e.g., lesions, stress-related biomarkers, and other clinical signs). Reduced well-being may negatively influence the pig’s health, growth, behavior, and emotional state.

Today, manual surveillance is generally conducted few times during the day in pig farms to visually screen for apparent signs of illness in their pigs, such as lethargy, lameness, and coughing [2]. Farm animal caretakers can know the extent of their pigs’ well-being with respect to their mental state (i.e., being calm, satisfied, relaxed, curious, playful, scared, stressed, or grunting) and physical state (i.e., healthy, medicated, or injured). However, human observations are subjective, as it is difficult to delineate the factors associated with the pig’s mental and physical state (e.g., in stress or pain) [2]. Manual surveillance may miss out to detect early signs of sickness (e.g., fever), particularly at night when the disease symptoms can be elevated. Moreover, it is difficult to give proper attention to all the animals and to identify subtle traits indicative of poor well-being in a timely manner. Without timely detection and intervention, the health of the pigs in a herd can be compromised, resulting in additional costs of medications [2], diagnostics [3], and therapy.

To complement manual surveillance, an automated behavioral assessment tool can be built that operates on a set of indicators to provide continuous surveillance, recognize stressful management practices, and detect early signs of sickness in individual pigs to promptly take corrective measures for the betterment of the animal and the entire population [1,4]. However, the identification of all relevant indicators is difficult because pig well-being is essentially a multidimensional and complex concept [5]. To accomplish automated behavioral assessment at the single animal resolution, a high-throughput data acquisition system is needed to measure the indicators noninvasively, under varying conditions, and in real time with minimal human intervention [6,7]. Furthermore, data science approaches are needed to identify relative value and relationships amongst the indicators [8,9].

Within the category of wearables, a variety of smart collars are commercially available that incorporate motion sensors, cameras, and/or microphones to record the daily activities of animals (e.g., dogs, cats, cattle, laboratory mice, sheep, goats, monkeys, marine life, and avian) [10]. The smart collars gather information about the physical location, movement, body temperature, grazing, and drinking behavior of these animals (Table 1). Most smart collars have an accompanying smartphone app through which the user can visualize, store, and analyze the gathered data [4]. In addition to smart collars, smart capsules (also called video endoscopy capsules) are used to visualize the gastrointestinal tract for disease diagnosis in a minimally invasive manner (e.g., Olympus EndoCapsule 10 System^TM^ and Medtronic PillCam SB 3 System^TM^). Moreover, in vitro microchip technologies have been custom designed for several applications in veterinary parasitology, such as for screening the efficacy of anthelmintics and chemical compounds against nematodes and enteroparasites for the pig host often tested by larval migration assays or fecal egg counts [11,12,13,14]. Imaging and/or thermal cameras have been used to record the movement and body heat emission of pig populations, along with subsequent machine learning analysis of recorded data [7,9]. Farm automation equipment is routinely deployed to control the indoor ventilation, feeding and watering, housing units, and thermal control systems (with regulated heating and cooling).

Most of the available technologies in human wearables and animal health monitoring are not feasible for direct adoption by the pig industry. For example, the wearables for human healthcare (e.g., smartwatches and fitness trackers) are cost prohibitive and priced over $50 per device. The anatomic conformation and behavior of the pigs make it virtually impossible to design wearables that can be used for extended periods of time (hours/days or more). Furthermore, skin-worn adhesive sensors are not suited for hairy pigs [15]. The commercialized smart collars designed for cows and companion animals are very expensive for monitoring a large number of pigs [10,16]. Moreover, the smart collars for cows and sheep are too large and heavy to be adopted for small piglets, while the electronic pet neck collars can be easily damaged because of the chewing behavior of pigs. Most sensor systems in pig farms (such as imaging cameras, microphones, climate control) cater to herd-level monitoring and do not have the resolution to pick up vital signs of individual pigs.

Aside from sensing and control units, there is a dearth of swine behavioral data to generate machine learning models that stand validated through multiple checks. The tools for gathering field data from swine farms, storing the acquired data, and applying predictive analytics are still premature but are constantly improving [9,17,18]. One could look for inspiration within the field of medical diagnostics, where there is a growing trend in low-cost, easily accessible, and field-deployable technology platforms for remote patient monitoring and decision support. In this regard, smartphone-based tools have been demonstrated for the objective assessment of physical attributes for clinical decision-making that could be adapted for swine behavioral monitoring, such as smartphone-based tools for the visual grading of pelvic asymmetry in equines [19] and skin cancer diagnostics in humans [20]. In addition to building a reliable data collection and data communication pipeline, there is a need to investigate new paradigms and models in swine health and welfare assessment that are fundamentally more robust and intelligent than the traditional models designed to estimate the future value of the animal (e.g., body mass, growth rate, genetic or breeding parameters).

**Figure 1:**
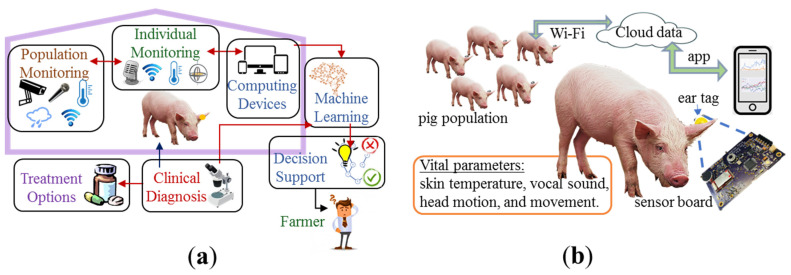
Overall theme for behavioral health monitoring in pig farms: (**a**) the pig farm is equipped with technologies to monitor individual animals and populations with mobile devices, data computing and storage units, machine learning, decision support tools, clinical diagnosis, and treatment options; (**b**) our measurement setup is shown. The ear tag with sensor board is attached to the ear lobe of individual pigs to measure vital parameters and send the data through a smartphone app to the cloud.

Our motivation lies in the objective assessment of swine behavior using a comprehensive set of indicators. The behavioral indicators should be meaningful and realistic, easy to collect on the farm in a noninvasive and unobtrusive manner, reliable to predict wellness levels, scalable to different animal classes (i.e., group size, growth phase), integrative with on-farm decision tools, and easily validated through alternate behavioral assessment tools [5,21]. In this paper, we demonstrate a proof-of-concept data acquisition system, based on a cluster of electronic sensors, to collect data on behavioral attributes on individual pigs (Figure 1). In Section 2, we describe the sensors to monitor external body temperature, physical movement, and vocalization of individual pigs. In Section 3, we show the results obtained from characterizing the sensors in the laboratory and tests from animal studies. In Section 4, we present a discussion on possible machine intelligence methodology to identify normal versus abnormal behavioral attributes in pigs, along with behavioral indicators that provide information about the pigs’ well-being. We discuss the challenges, limitations, and path forward for the development and dissemination of this technology, including the social–economic–ethical considerations for technology adoption.

## 2. Materials and Methods

### Electronic Sensor Boards

**Figure 2:**
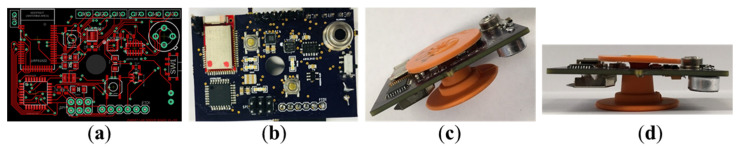
Prototype design and assembly: (**a**) CAD layout of the electronic sensor board; (**b**) the fabricated PCB with soldered electronic chips; (**c**,**d**) inclined view and side view of an assembled sensor board sandwiched between the orange-colored ear tag.

One cluster of sensors (soldered on a custom printed circuit board (PCB)) was attached to an ear tag for pigs to record parameters such as external temperature, head tilt, movement, and vocalization. A Bluetooth-enabled, application-specific integrated circuit (ASIC) chip was soldered on the electronic sensor board for the wireless transmission of raw data to a computing device located in or nearby the pen. A replaceable lithium battery was housed within a battery holder on the board. Figure 2 shows the computer-aided design (CAD) software layout of the electronic sensor board, fabricated PCB, and its attachment to a sample ear tag. The physical dimensions of the electronic sensor board were 3.05 cm × 3.05 cm × 1.27 cm (L × W × H), with a weight of 5 g and a development cost of around USD 20 per sensor board.

The electronic sensor board had the following sensors:

(i) A contactless, infrared temperature sensor (MLX90614) to measure both the ambient temperature and external body temperature of the pig;
(ii) A 3-axis accelerometer (ADXL345) and gyroscope (ADXRS300) to record data on head tilt and movement, which is correlated to aggression, lethargy, lameness, or neurological disorders;
(iii) A sound sensor (MAX9814) to capture the vocalizations for the identification and classification of call types or aggressive behavior;
(iv) A Bluetooth low energy (BLE) module (NRF51822 SoC) to receive data from the abovementioned sensors at a preselected rate (e.g., 5 samples per second). This BLE module incorporates a chip antenna, 32-bit ARM Cortex M0 CPU, 256 kB Flash Memory/16 kB RAM, and 32 GPIO. The BLE module is suited for ultralow-power wireless communication at 2.4 GHz by supporting third-party I2C, SI, UART, and PWM interfaces.

A 3.3 V, 230 mAh coin cell battery (CR 2032) provided power to the sensors and BLE module on the PCB. The electronic sensor boards were sealed in polymeric coatings (e.g., SU-8, PDMS) before being put within ear tags.

## 3. Results

### 3.1. Laboratory Testing

All the sensors and the Bluetooth module were tested in our device characterization laboratory according to the manufacturer’s specifications. The software development kit (SDK) from Nordic Semiconductor was downloaded (nRF5 SDK v12.3.0) prior to testing the Bluetooth module. We prepared 10 electronic sensor boards. Within every board, data from each sensor were read by placing the board next to the receiver or at a certain distance (roughly 1 m). We tested different sampling rates (from five samples per second to one sample per 10 min) and sleep times of the sensors (1 s to 10 min) to maximize battery life while still having adequate data resolution. With a sampling rate of five samples per second, the Bluetooth module and gyroscope consumed the maximum current (6 mA), followed by the sound sensor (3.1 mA), temperature sensor (1.5 mA), and accelerometer (0.14 mA). Assuming that the CR2032 had a capacity of 230 mAh, this translates to a battery life of 13.74 h. We found that the battery life as built was increased to over 3 days by prolonging the sampling rate and sleep time. The battery life can be extended by incorporating adaptive sampling and power management schemes.

### 3.2. Animal Testing

**Figure 3:**
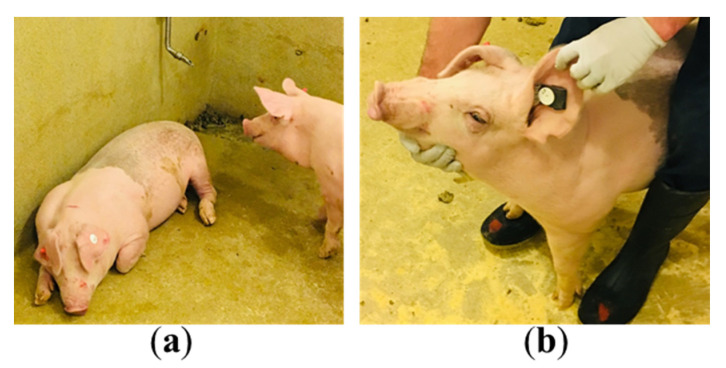
Animal testing: (**a**) two growing pigs were used for testing the electronic sensor boards; (**b**) the ear tag with the sandwiched electronic sensor board was attached to the left ear of the pig. The sensors and ASIC chip were remotely controlled by predefined awake/sleep cycles.

**Figure 4:**
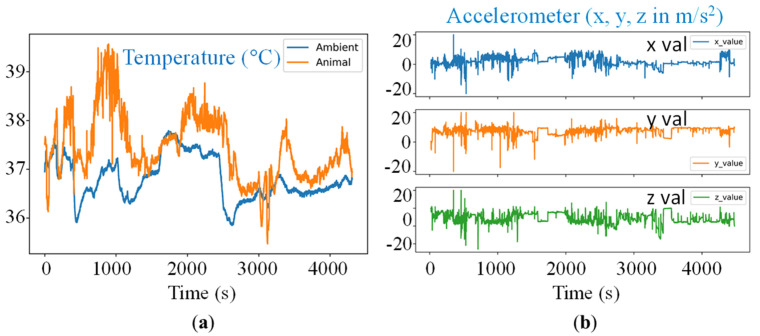
Time-series data recorded from pig testing using the electronic sensor board. Sample data from the animals is shown here as obtained from the on-board (**a**) temperature sensor and (**b**) 3-axis accelerometer. The sound sensors collected the baseline curves, but there were no useful events (e.g., coughing or screaming) during the recorded time period.

For animal testing, the electronic sensor board was sandwiched between the two pieces of the ear tags and attached to the ear of pigs. Tests were conducted on two growing pigs (body weight: around 18 kg) (Figure 3). We did not observe any unnatural behavior from the pigs during or after mounting the electronic sensor boards on the ear. The data from the growing pigs were recorded for around 4500 s or 75 min (Figure 4). The animal tests confirmed that remote data collection from the sensor boards was near real time and fully automated over a distance of 15.25–18.3 m.

## 4. Discussion

### 4.1. Machine Intelligence for Swine Behavioral Health Monitoring

**Figure 5:**
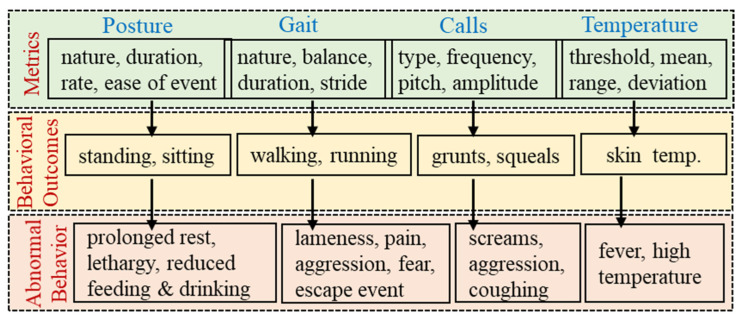
Flowchart for metrics, behavioral outcomes, and indicators of poor well-being.

A single behavioral health indicator can misjudge the well-being status, and therefore, a realistic set of indicators are required for accurate assessment [22]. In this context, machine intelligence is valuable to assimilate and process data from multiple sensors and human observations for subsequent decision support [8,23]. Currently, our data collection activities are underway with the intent to generate sufficiently large datasets to map the raw sensors’ data into predictable behavioral traits, as described in Figure 5. Here, the activity sensors measure the raw data related to posture and gait (e.g., the nature, duration, rate, and ease of events). Thereafter, machine intelligence based on linear regression will be used to analyze the raw data and distinguish different postures and gaits. The sound sensors measure vocalization in terms of the call duration, call rate, main frequency, peak amplitude, and peak frequency. This will help to distinguish the nature and type of call. Abnormal behavioral traits could include prolonged rest, reduced eating and drinking, lameness, pain, aggression, escape events, screams, coughing, and fever. These abnormal traits could be identified by machine learning algorithms such as random forest, decision tree, K-nearest neighbor, support vector machine, artificial neural networks, naïve Bayes, linear regression, logistic regression, and gradient boosting.

**Figure 6:**
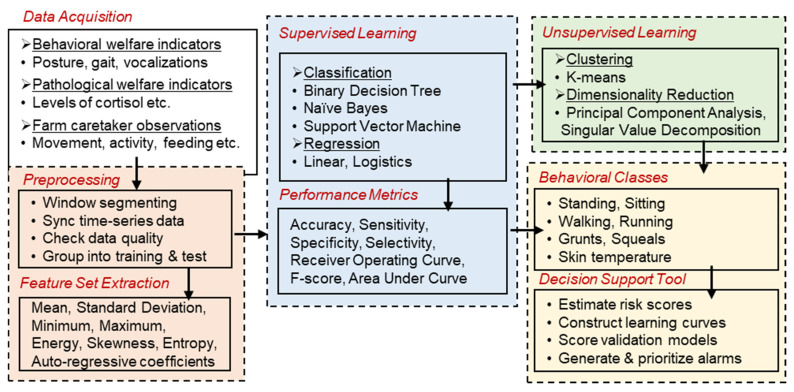
Process flow during machine intelligence involves data acquisition, preprocessing, feature set extraction, supervised or unsupervised learning, class definitions, performance metrics, and decision support.

One suggested scheme for processing the raw data from different electronic sensors is shown in Figure 6. The data processing starts by segmenting the incoming sensors’ data into separate time windows, as described in our previous work on deep learning [24]. We compute a set of features that give a good spectral and temporal representation of the data (such as mean, standard deviation, energy, skewness, entropy, and auto-regressive coefficients). Next, we employ classifiers on the computed set of features. When a feature exceeds a certain threshold, the behavior is categorized by a classifier into one of the behavioral classes, and an alert is sent to the farmer. The choice of behavioral traits to train machine learning models should be those that are most relevant to the pig farmer. Some examples of behavioral classes related to pig activity are walking, standing, sitting, lying, head down, grunting, screaming, feeding, and drinking. Choosing an appropriate threshold level is often beneficial, as a low threshold leads to many false positives and false alerts, while a high threshold gives many false negatives and missed true events [17,25]. In most cases, the choice of the optimal threshold for a classifier depends on the datasets, operating conditions, type of model used, receiver operating characteristic (ROC) curve, precision-recall curve, and the intuitive sense of the model developer.

Table 2 lists the hardware and software features, and potential benefits of our ear tag sensor board for the pig farmer. The technical features are designed to be modular and easily upgradable to support autonomous and remote monitoring of behavioral health indicators. There are near-term challenges during technology development, such as establishing validity with large amounts of real-world data from experimental and field settings. Building reliable machine learning models and decision support tools for large-scale pig farms is also nontrivial [6,26,27]. False alarms increase costs, reduce the trust of operators, and decrease efficiency. Thus, it is advisable to identify farm situations of high prevalence or priority to the farmer where autonomous monitoring and alert notification will complement or supersede manual surveillance [5,27].

### 4.2. Roadmap for Technology Adoption

For technology adoption, bringing the pig farmers on board to test the ear tag wearable technology is critical but challenging. The pig farmer may have misconceptions about digital technologies with respect to their perceived relative value, complexity, adaptability, and compatibility [10,16]. Table 3 lists the inducements and impediments related to technology adoption of the presented ear tag sensor boards.

Technology adoption is often facilitated by communicating its role in promoting the overall well-being of human–animal–environment [1,10]. In this regard, the ethical issues raised by the ear tag sensor boards can be assessed by a two-dimensional bioethics matrix, originally proposed by Dr. Ben Mepham (Special Professor in Applied Bioethics, University of Nottingham) for food biotechnologies [28,29].

In the bioethics matrix, as shown in Table 4, there are three prima facie principles: respect for well-being, respect for autonomy, and respect for justice/fairness. There are five principal stakeholders whose interests are implicated—pigs, pig farmers, consumers, the public at large, and the environment. The matrix framework and components are based on the original work by Dr. Ben Mepham [28,29]. On the one hand, the pork industry is evolving to make the swine production process more rapid and more profitable with minimal human–pig interaction. On the other hand, consumers prefer meat from happy and healthy animals, while the public at large wants transparency in information about animal well-being and handling during production [30]. Respect for the well-being of pigs is achieved by avoiding causes of pain or harm, improving health or welfare, and mitigating risks or costs [31]. Respect for autonomy ensures freedom of choice and behavior for the pigs. Respect for fairness or justice refers to norms regarding fair distribution of costs, risks, and benefits for the animals to protect the intrinsic value of the pigs. The well-being of the pig farmer is reached by satisfactory profits and improved working conditions, autonomy by having the freedom to manage actions, and fairness by access to fair trade deals. Farmers who have more access to data about the health and well-being of their pigs may be expected to benefit and should have increased opportunities to intervene to promote the health and welfare of their animals. However, pig farmers may feel pressurized by the politics and media and skeptical of participating in welfare programs [32]. Modern consumers are interested in the quality and safety of their food, as well as in the humane treatment of animals raised for slaughter and the environmental sustainability of the food they consume [33]. The public at large, and even those who do not consume pork products, may have an interest in the process of pork production and improving the health and safety of pork production facilities. Pork production involves risks to the environment that must be taken into account in the production process, and wherever possible, environmental risks should be minimized [31].

Social studies can help understand the pig farmers’ behavior, existing farm practices, sociocultural perceptions, and decision-making methods while communicating the realistic deliverables [1,16]. Through open discussions and social studies with the potential users, it is possible to understand the factors that influence their decisions on adopting the presented wearable technology. As with other new technologies, the user inputs will help identify effective ways to educate them about the technology’s merits and risks from different sociocultural and economic perspectives. By demonstrating the technology at work and rationalizing its cost-versus-benefit analysis in video format and simple language, it may be possible to slowly gain the trust of potential users and ensure that our technology makes its way from the laboratory to the real world.

However, the window for voluntary adoption by hog producers may be fast closing. Animal welfare and food safety is an increasingly important part of purchasing decisions by consumers. For the former, consumers are insisting on minimal welfare standards [34,35], while the latter is centered around consumer concerns over antimicrobial resistance in meat [36,37]. Concentration in food wholesale and retail means consumer pressure can be brought to bear on a few firms that have large market power [38]. As consumers demand more stringent animal welfare and antibiotic use rules, wholesalers and retailers will likely force changes down the supply chain onto livestock producers. In short, the adoption of intelligent sensing systems to improve animal health and reduce antibiotic use may become an economic necessity for hog producers to stay in business.

Additionally, the devastation wrought by coronavirus disease 2019 (COVID-19) highlights how improving animal health is an important part of promoting public health and resilience to pandemics. Large-scale hog production provides opportunities for the generation and transmission of novel viruses from hogs to humans [39]. Although rare, certain variants of swine influenza could emerge to become a regional epidemic, if not a pandemic [40]. Workers in hog production and processing are at risk of contracting these novel viruses and may transmit the disease to the broader community [41]. Thus, early detection of diseases in large-scale swine production facilities is more important than ever in the light of COVID-19.

On the technology development front, there are possibilities to expand the breadth of sensing functionalities tailored to the needs and expectations of the swine farms. The electronics sensors and communication systems are constantly evolving, and there are many options for low-cost and high-performance sensors manufactured by various semiconductor companies [42,43]. Therefore, in conjunction with the presented electronic sensor board, there is scope to equip the farms with other types of farm deployable sensors in swine farms. For instance, ambient and environmental sensors can be installed to monitor different locations in the barn and transmit data to the local server via Bluetooth modules. These sensors can record environmental parameters in the barn such as humidity, room temperature, airflow, and levels of common gases. Some examples of commercial environmental sensors that can be used in pig farms are the capacitive humidity sensor (DHT11 sensor, Adafruit Industries, New York City, NY, USA) to report the dampness estimate in the room, miniaturized airflow transducers (Kanomax^TM^ Model 0965-01, Kanomax USA Inc., Andover, NJ, USA) to measure the air velocity and indoor airflow, wireless gas sensors to monitor the levels of CO_2_, CH_4_, H_2_S, and NH_3_ (Monnit^TM^, Agrologic^TM^), and infrared cameras (FLIR^TM^) and webcams (Pig Cam^TM^) to take images of the pigs.

There are limitations in the ear tag sensor-board technology presented here. Firstly, the current development cost of the electronic sensor boards (approximately USD 20 to USD 30 per board) is a significant investment for swine producers in the short term, even though these reusable boards may have a justifiable return on investment in the long term. Secondly, extending the battery life of wearable devices is a challenge considering the high-power consumption for BLE communication and inaccessibility to recharge or replace the batteries during animal testing. Thirdly, there is a risk of damage to the sensor boards and loss of data communication while the boards are mounted on the pigs. The dimensions of the sensor board are greater than the ear tags used in our tests, which may interfere with the pigs. Using larger ear tags or reducing the size of the sensor boards would be advisable. Fourthly, there are realistic limits on the number of behavioral patterns that can be accurately characterized by machine intelligence. Additionally, one set of machine learning classifiers may not work on different herds and different farms, and some degree of customization may be required. Lastly, winning and retaining the trust of active users is challenging, as there can be considerable variability in user expectations and the performance of decision-making tools for different pig farms.

## 5. Conclusions

Intelligent sensing systems have the potential to automate and integrate various activities in swine production, such as breeding, feeding, handling, housekeeping, and disease management. However, the pace of technology adoption in pig farming is slow because of three main technological deficiencies—lack of adoptable sensor technologies, limited availability of meaningful data, and unproven means to translate the data science into actionable items. Our work here was primarily motivated to address the first deficiency. We showed a proof-of-concept working of an electronic sensor board that can be attached to the ear tags of individual pigs to record physical activity. Aside from the components used in our board, there are several other options for off-the-shelf sensors and communication modules that can be purchased and tested depending on their functionality, features, compatibility, physical dimensions, and pricing. We recognized a number of inducements and impediments for its user acceptance as a reliable behavioral assessment tool. For technology adoption, one key hurdle lies in collecting and analyzing massive quantities of field data with cross validation by farm animal caretakers. This is a prevalent challenge for the wearables industry at large and requires cooperative efforts from all stakeholders, from swine producers to pork consumers.

## Figures and Tables

**Table 1:** Commercialized products for tracking humans, pets, and livestock.

Products	Description
1. Tractive, FitBark, Whistle 3	Real-time GPS activity tracker for cats and dogs
2. TekVet, FeverTag	Temperature sensor for livestock
3. Abilify, EndoCapsule EC-10, Vital Herd	Ingestible pill to track core, heart rate, respiration rate, pH levels, drug doses
4. Cowlar, CowCollar, Cattle Watch	Electronic collar for cows to track body motion, cud-chewing, location, create invisible fence
5. E-Shepherd	Electronic collar for sheep to generate irritating sound and lights signals to deter predators
6. Apple Watch, Fitbit, Garmin, Samsung Gear	Track fitness, performance, heart rate, respiration, stress, blood oxygen levels, sleep quality

**Table 2:** Attributes and benefits of the ear tag sensor board for the pig farmer.

Attributes	Benefits of the Ear Tag Sensor Board
Hardware features	Light weight, long battery life, low cost, wireless capability, easily upgradable, easy programming, modular, reusable, safe
Software features	Ease of use, multiuser access, autonomous, remote operations, real-time data acquisition and management, decision support
Potential benefits	Real-time awareness of pig vital signs, early disease detection, timely isolation of diseased animal from the herd, cross-platform data compatibility, upgradable and customizable platform, data transparency and privacy, reduced labor, increased profits
Challenges during development	Scope of clinical trials, safety, and health regulations, sustained technical improvements, fluctuations in market trends and surveillance technologies, user acceptability and trust

**Table 3:** Perceived inducements and impediments in the adoption of ear tag sensor boards by pig farmers.

Adoption Factors	Inducements and Impediments
Technical factors	Learning curve, automation needs, safety, ethics, maintenance support, data accessibility and privacy, trust in decision support
Existing farming practices	Management practices, herd size, labor and service inputs, surveillance techniques, managerial skills, clinical access
Sociocultural factors	Trust groups, local practices, community size, farm size, intent of rearing, labor constraints, digital literacy, communication modes
Economic factors	Cost–benefit clarity, competitiveness, fluctuating markets, financing options, income growth, profit margin, wealth, supply and demand
Health factors	Control over disease outbreaks, early illness detection, reliability of health data, clinical validation, treatment options
External factors	Government and trade policies, farming regulations, transportation, environmental legislation, carbon footprint, bioethics

**Table 4:** Bioethics matrix for developing swine welfare assessment tools.

Respect for	Well-Being	Autonomy	Fairness
Pigs	Avoid pain, improved welfare	Behavioral freedom	Intrinsic value
Farmers	Satisfactory profit, better work condition	Managerial freedom of action	Fair trade laws and practices
Consumers	Better quality of life, food safety	Democratic, informed choices	Availability of affordable pork
The Biota	Conservation	Biodiversity	Sustainability

Source. Motivated and adapted from the pioneering works of Dr. Ben Mepham [28,29].
